# A large population-based study of the mental health and wellbeing of children and young people in the North of England

**DOI:** 10.1177/1359104520925873

**Published:** 2020-06-03

**Authors:** Barry Wright, Megan Garside, Victoria Allgar, Rachel Hodkinson, Helen Thorpe

**Affiliations:** 1 University of York, UK; 2 Leeds and York Partnership NHS Foundation Trust, UK; 3 Rotherham Doncaster and South Humber NHS Foundation Trust, UK

**Keywords:** Children, mental health, school, well-being, education, survey

## Abstract

**Background:**

There has been a recent reported rise in prevalence of mental health problems among children in the United Kingdom, alongside increased referrals into specialist services. There is a need for up-to-date information regarding changing trends of young people’s mental health to allow for improved understanding and service planning.

**Objectives:**

This article aims to provide an overview of the current mental health and well-being of years 8, 9 and 11 secondary school–aged pupils from two large regions in the North of England.

**Method:**

This was a cohort cross-sectional study. Measures including the Strengths and Difficulties questionnaire, the EQ-5D-Y, social media use questions, and a mental health service use questionnaire were completed by participants.

**Results:**

In total, 6328 questionnaires were returned from 21 secondary schools. One in 10 participating pupils scored ‘very high’ for total mental health difficulties. Significant differences on well-being scores were found between both gender and year groups.

**Conclusion:**

In recent years, the proportion of children facing mental health problems has increased. In particular, high levels of female pupils and year 11 pupils report facing difficulties. It is important to develop targeted, accessible interventions, and to continue to collect up-to-date measures for this population.

## Introduction

Approximately 50% of mental health problems are thought to begin before the age of 14 years (Kessler et al., 2005). Poor mental health has been linked to associated poor outcomes, such as lower educational achievement, substance use and poor physical health (Naylor et al., 2016; Prince et al., 2007). Preventive measures to address mental health early in life is proposed to improve outcomes for young people and lead to more positive outcomes later in life (Wellander et al., 2016).

Recent reports have identified an increase in the rates of mental health problems in young people over time (Fink et al., 2015; Murphy & Fonagy, 2013; Rutter, 1989). The latest Office of National Statistics results (Sadler et al., 2018) report a prevalence of mental disorders among young people in the United Kingdom of 14.4% (11–16 year olds), an increase on the previously reported prevalence of 11.7% (11–16 year olds) (McGinnity et al., 2005).

This reported increase has come against a backdrop of increasing mental health morbidity, inequalities and referrals into specialist Child and Adolescent Mental Health Services (CAMHS) (NHS Benchmarking Network, 2016; Frith, 2016) as well as an increase in counselling support needed from services such as ChildLine, and a recent increase in schools seeking referrals for professional mental health help (National Society for the Prevention of Cruelty to Children, 2018a, 2018b). Several factors have been suggested as contributing to this rise, including poverty, societal changes brought about by advances in technology (including social media) and anxiety and pressure from the school environment (Evans & Hurrell, 2016; Hood & Waters, 2017; Pitchforth et al., 2019; Sweeting et al., 2010).

The initial ‘Future in Mind’ report (NHS England & Department of Health, 2015) and subsequent follow-up report (Burt, 2016) identified the need for improved emotional, psychological and mental health services for children and young people within schools. Both Local Authority (LA) and CAMHS budgets were reduced during the recent time of austerity (Young Minds, 2015). 6.7% of local-area mental health funding is spent on child services despite this being nearly 20% of the population (Children’s Commissioner, 2018). The children and young people’s mental health UK Government Green Paper (Department of Health and Social Care and Department for Education, 2017) identifies the need for effective interventions across a range of settings, including schools. It identifies children and young people’s mental health as a key Government priority, as they aim to deliver the objectives outlined in *Future in Mind*. Despite a clear increase in need for low-level interventions to support children with mental health difficulties, there is huge variation between local areas in terms of service provision and spending, and reports of nearly 60% of UK areas seeing LA spend per child decrease between 2016/2017 and 2018/2019 (Children’s Commissioner, 2019).

It is important to collect large-scale data on the mental health and well-being of children and young people, to understand the current prevalence and allow trends to be identified over time. Increased understanding enables effective future planning of services. This study aimed to provide a current overview of the mental health of young people attending secondary schools across two large regions in the North of England.

## Method

### Design

This large-scale evaluation is a cohort cross-sectional design and presents questionnaire data collected from children and young people attending secondary schools across the North of England during Spring of the 2017/2018 academic school year. Ethics approval was obtained from the University of York Health Sciences Research Governance Committee.

### Participants

A total of 6328 pupils participated from 21 mainstream secondary schools. Demographic information about participating pupils is presented in Table 1.

**Table 1.** Characteristics of respondents.

Characteristic	*n* (%)
Gender	
Male	2443 (39)
Female	2827 (45)
Prefer not to say/missing	1058 (17)
Year group	
8	2907 (46)
9	1711 (27)
11	1603 (25)
Missing	107 (2)

The study sample includes a diverse range of secondary schools, from both urban and rural areas, and across a range of socioeconomic areas. Approximate school sizes range from 100 to 2000 pupils. We included any mainstream secondary school, including community schools, free schools and academies. Two of the schools are Church of England, three are Roman Catholic and 16 are secular. The percentages of pupils eligible for free school meals varied, from approximately 2% to 20% and the reported number of pupils eligible for Pupil Premium ranged from approximately 80 to 400 pupils.

### Measures

Participating pupils completed the questionnaire booklets using an ID provided by the research team. Booklets did not include personal identifying details. They included the self-report 11–17 year olds Strengths and Difficulties Questionnaire (SDQ) (Goodman, 1997) with impact supplement, two brief bespoke questions regarding social media use, the EQ-5D-Y (Wille et al., 2010) and a bespoke service use questionnaire, developed by the research team. Details of pupils’ year group and gender were also collected.

The SDQ (Goodman, 1997) measures responses to 25 questions across five subscales. Four represent difficulties, including emotional problems, conduct problems, hyperactivity, peer problems and one represents a strength; prosocial behaviour. The first four subscales can be combined to give a total difficulties score. The scores on each subscale can be categorised as scoring ‘Close to Average’, ‘Slightly Raised’, ‘High’ or ‘Very High’. These thresholds are created from a UK-based population survey which selected bandings where 80% of children scored ‘Close to Average’, 10% scored ‘Slightly Raised’, 5% scored ‘High’ and 5% scored ‘Very High’. As only self-report measures were collected from this sample, it was not appropriate to categorise responses as scoring ‘unlikely’, ‘possible’ and ‘probable’ for a mental disorder (Goodman et al., 2000).

Two brief bespoke questions about social media use were included asking pupils to rate the effect social media had on their mood as either ‘positive’, ‘negative’ or ‘neither positive or negative’, and rating how much using social media impacted their mood over the past week, as either ‘not at all’, ‘some of the time’, ‘more than half of the time’ or ‘all of the time’.

The EQ-5D-Y (Wille et al., 2010) is the child-version of the EQ-5D quality-of-life measure. It measures responses on five dimensions; ‘mobility’, ‘looking after myself’, ‘doing usual activities’, ‘having pain or discomfort’ and ‘feeling worried, sad or unhappy’. The EQ visual analogue scale (EQ-VAS) was included, asking participants to rate their health on a scale, from ‘the best health they can imagine’ to ‘the worst health they can imagine’.

The bespoke service use questionnaire developed by the research team allowed pupils to record what physical health, school-based and mental health services they have used in the past 12 months.

### Procedure

Four LAs were contacted and asked to distribute study information to secondary school staff. In January 2018, the research team then contacted all eligible schools to further explain the study and offer participation. Schools were eligible if they were a mainstream secondary school, no specialist schools or primary schools were included. Of approximately 84 eligible schools, one withdrew due to capacity issues, and approximately 12 actively decided not to participate; reasons included capacity issues, staff changes and competing demands due to participation in another survey. The other schools were contacted several times and did not respond within the recruitment timeframe.

Year 8 (12–13 years old), year 9 (13–14 years old) and year 11 (15–16 years old) pupils at consenting schools were given participant information sheets about the study and were able to choose not to participate by completing and returning an opt-out form, signed by their parents/guardians. The school also notified parents/guardians of the study and the opt-out form by email or text. Children could also opt-out on the day and choose not to complete the questionnaire. Pupils completed questionnaires at school in Personal, Social & Health Education lessons or form time during the Spring Term.

Questionnaires were collected by the research team and data inputting and analysis then took place. Participating schools were kept anonymous and pupil questionnaires were assigned an alphanumeric ID code to ensure confidentiality. All participants were informed that no individual pupils’ scores could be identified. A list of support services (including school contacts and local child mental health services) was given to each pupil in a separate information leaflet.

In total, 6328 questionnaires were returned from 21 schools from a total cohort of approximately 9896 eligible pupils, achieving a response rate of 64%.

The raw scores from questionnaires were entered onto a secure database. To ensure data entry reliability, 5% of the whole data set (*N* = 332) were randomly selected and cross checked by a research assistant. No systematic errors were found. For any school that had more than one error, 10% of the questionnaires from the whole school were checked. If further errors were then found, all questionnaires from this school were re-entered.

### Analysis

Summary statistics are presented as *M* (*SD*), median (IQR) and *n* (%). To compare scores by gender, a Mann–Whitney test was used and a Kruskal–Wallis test was used to compare age groups. Chi-square tests were used to compare the categories of SDQ by gender and year group. A *p*-value of <.05 was considered to indicate statistical significance. All analyses were undertaken on SPSS (IBM Corp.; Released 2017. IBM SPSS Statistics for Windows, Version 25.0. Armonk, NY, USA).

## Results

### SDQ

The self-report SDQ with impact supplement (for ages 11–17 years) was completed by 6328 secondary school pupils. Table 1 shows the sample demographic characteristics.

### Overall scores

Table 2 shows the summary of the pupil scores for the self-completed SDQ (Goodman et al., 2000). Overall, 9.4% (95% confidence interval [CI] = [8.7%, 10.1%]) scored ‘very high’ on total difficulties. This score combines responses on each of the difficulty subscales (emotional, conduct, hyperactivity and peer problems). This shows that 1 in 10 pupils score ‘very high’ for total difficulties on the SDQ, which could suggest they are at a higher risk for developing a mental disorder. 15% of all pupils scored within the ‘very high’ and ‘high’ ranges for total difficulties on the SDQ.

**Table 2.** Summary scores.

	*M* (*SD*)	Median (IQR)	Average	Slightly raised	High	Very high	*n*
Total difficulties	11.3 (5.9)	11 (7–15)	4571 (72%)	762 (12%)	379 (6%)	593 (9%)	6305
Emotion	3.4 (2.5)	3 (1–5)	4310 (68%)	629 (10%)	510 (8%)	866 (14%)	6315
Conduct	1.8 (1.8)	1 (0–3)	5270 (84%)	474 (8%)	302 (5%)	267 (4%)	6313
Hyperactivity	4.1 (2.5)	4 (2–6)	4561 (72%)	620 (10%)	455 (7%)	674 (11%)	6310
Peer problems	1.9 (1.7)	1 (1–3)	4554 (72%)	788 (13%)	457 (7%)	511 (8%)	6310
Prosocial	7.0 (2.0)	7 (6–8)	3909 (62%)	950 (15%)	757 (12%)	707 (11%)	6323
Impact	0.7 (1.5)	0 (0–1)	4351 (70%)	723 (11%)	448 (7%)	687 (11%)	6209

SD: standard deviation; IQR: interquartile range.

There was a high percentage of pupils scoring ‘very high’ on the emotional subscale (14%). There was a low percentage of pupils scoring within the ‘very high’ range on the conduct subscale (4%), with a higher proportion of pupils scoring within the ‘average’ range for this subscale (84%).

### Gender

Results from a Mann–Whitney test are presented in Table 3. These results show that there were statistically significant differences between males and females for all subscales except peer problems.

**Table 3.** Scores by gender.

	Male			Female			*p*-value
	*M* (*SD*)	Median (IQR)	*n*	*M* (*SD*)	Median (IQR)	*n*	
Total difficulties	10.5 (5.7)	10 (6–14)	2436	11.6 (5.9)	11 (7–15)	2820	<.001
Emotion	2.5 (2.1)	2 (1–4)	2438	4.3 (2.5)	4 (2–6)	2824	<.001
Conduct	2.0 (1.8)	2 (1–3)	2437	1.5 (1.6)	1 (0–2)	2823	<.001
Hyperactivity	4.2 (2.5)	4 (2–6)	2437	3.9 (2.5)	4 (2–5)	2823	.004
Peer problems	1.8 (1.6)	1 (1–3)	2438	1.8 (1.7)	1 (1–3)	2821	.976
Prosocial	6.4 (2.0)	6 (5–8)	2439	7.6 (1.7)	8 (7–9)	2827	<.001
Impact	0.5 (1.2)	0 (0–0)	2397	1.0 (1.7)	0 (0–1)	2793	<.001

SD: standard deviation; IQR: interquartile range.

Table 4 shows that females were more likely to have very high scores than males for total difficulties (10% vs 7%), emotional difficulties (21% vs 6%) and impact scores (15% vs 7%). Male pupils were more likely to have very high scores than female pupils for conduct problems (5% vs 3%), hyperactivity (11% vs 9%) and prosocial difficulties (17% vs 5%).

**Table 4.** Categories by gender.

	Gender	Average	Slightly raised	High	Very high	*p*-value
Total difficulties	Male	1875 (77%)	259 (11%)	120 (5%)	182 (7%)	<.001
	Female	1968 (70%)	380 (13%)	188 (7%)	284 (10%)	
Emotion	Male	2009 (82%)	172 (7%)	122 (5%)	135 (6%)	<.001
	Female	1573 (56%)	338 (12%)	308 (11%)	605 (21%)	
Conduct	Male	1988 (82%)	208 (9%)	121 (5%)	120 (5%)	<.001
	Female	2465 (87%)	181 (6%)	103 (4%)	74 (3%)	
Hyperactivity	Male	1739 (71%)	246 (10%)	186 (8%)	266 (11%)	.019
	Female	2123 (75%)	246 (9%)	186 (7%)	268 (9%)	
Peer problems	Male	1755 (72%)	312 (13%)	188 (8%)	183 (8%)	.516
	Female	2070 (73%)	333 (12%)	199 (7%)	219 (8%)	
Prosocial	Male	1186 (49%)	447 (18%)	399 (16%)	407 (17%)	<.001
	Female	2142 (76%)	331 (12%)	204 (7%)	150 (5%)	
Impact	Male	1865 (78%)	244 (10%)	131 (5%)	157 (7%)	<.001
	Female	1783 (64%)	365 (13%)	231 (8%)	414 (15%)	

### Year group

The results of a Kruskal–Wallis test are presented in Table 5. This shows a significant difference between year groups on each of the SDQ subscales, except for the conduct subscale. Table 6 shows year 11 pupils were more likely to score within the ‘very high’ range than year 8 and year 9 pupils on the emotional problems subscale (18% vs 12% vs 13%) and on the peer problems subscale (14% vs 10% vs 11%).

**Table 5.** Scores by year group.

	Year 8			Year 9			Year 11			*p*-value
	*M* (*SD*)	Median (IQR)	*n*	*M* (*SD*)	Median (IQR)	*n*	*M* (*SD*)	Median (IQR)	*n*	
Total difficulties	11.1 (6.0)	10 (7–15)	2898	10.8 (5.8)	10 (6–15)	1708	12.0 (5.7)	11 (8–16)	1594	<.001
Emotion	3.3 (2.4)	3 (1–5)	2902	3.3 (2.5)	3 (1–5)	1710	3.8 (2.6)	4 (2–6)	1597	<.001
Conduct	1.9 (1.8)	1 (0–3)	2902	1.8 (1.7)	1 (0–3)	1709	1.8 (1.7)	1 (0–3)	1596	.232
Hyperactivity	4.1 (2.5)	4 (2–6)	2901	4.1 (2.5)	4 (2–6)	1709	4.3 (2.4)	4 (2–6)	1594	.001
Peer problems	1.8 (1.7)	1 (1–3)	2900	1.7 (1.6)	1 (1–3)	1709	2.0 (1.7)	2 (1–3)	1596	<.001
Prosocial	7.1 (1.9)	7 (6–9)	2905	6.9 (1.9)	7 (6–8)	1710	6.8 (2.0)	7 (5–8)	1601	<.001
Impact	0.7 (1.5)	0 (0–1)	2845	0.7 (1.4)	0 (0–1)	1692	0.9 (1.7)	0 (0–1)	1575	<.001

SD: standard deviation; IQR: interquartile range.

**Table 6.** Categories by year group.

	Year group	Average	Slightly raised	High	Very high	*p*-value
Total difficulties	Year 8	2121 (73%)	352 (12%)	151 (5%)	274 (9%)	.001
	Year 9	1280 (75%)	177 (10%)	100 (6%)	151 (9%)	
	Year 11	1097 (69%)	219 (14%)	119 (7%)	159 (10%)	
Emotion	Year 8	2032 (70%)	294 (10%)	226 (8%)	350 (12%)	<.001
	Year 9	1203 (70%)	169 (10%)	113 (7%)	225 (13%)	
	Year 11	998 (62%)	156 (10%)	162 (10%)	281 (18%)	
Conduct	Year 8	2387 (82%)	231 (8%)	145 (5%)	139 (5%)	.163
	Year 9	1450 (85%)	116 (7%)	80 (5%)	63 (4%)	
	Year 11	1355 (85%)	114 (7%)	69 (4%)	58 (4%)	
Hyperactivity	Year 8	2121 (73%)	283 (10%)	196 (7%)	301 (10%)	.402
	Year 9	1249 (73%)	154 (9%)	123 (7%)	183 (11%)	
	Year 11	1119 (70%)	171 (11%)	124 (8%)	180 (11%)	
Peer problems	Year 8	2103 (73%)	346 (12%)	212 (7%)	239 (8%)	.022
	Year 9	1276 (75%)	205 (12%)	110 (6%)	118 (7%)	
	Year 11	1103 (69%)	224 (14%)	125 (8%)	144 (9%)	
Prosocial	Year 8	1881 (65%)	427 (15%)	317 (11%)	280 (10%)	<.001
	Year 9	1043 (61%)	266 (16%)	206 (12%)	195 (11%)	
	Year 11	926 (58%)	238 (15%)	219 (14%)	218 (14%)	
Impact	Year 8	2019 (71%)	306 (11%)	211 (7%)	309 (11%)	.004
	Year 9	1220 (72%)	202 (12%)	108 (6%)	162 (10%)	
	Year 11	1048 (67%)	197 (13%)	123 (8%)	207 (13%)	

### EQ-5D-Y

The EQ-5D-Y measures pupils’ responses on five dimensions. The response options range from ‘no problems’ (which was coded as 1 for scoring), to ‘some problems’ (coded as 2) and ‘a lot of problems’ (coded as 3) for the first four questions. For the final question, the response options range from ‘not worried, sad or unhappy’ (coded as 1), to ‘a bit worried, sad or unhappy’ (coded as 2) and ‘very worried, sad or unhappy’ (coded as 3). The percentage of pupils responding as having either ‘a lot of problems’ or feeling ‘very worried, sad or unhappy’ were calculated for each dimension (Table 7).

**Table 7.** EQ-5D.

	Score	Total	Males	Female	*p*-value	Year 8	Year 9	Year 11	*p*-value
Mobility	1	5633 (91%)	2204 (92%)	2550 (91%)	.156	2547 (90%)	1539 (91%)	1464 (93%)	.008
	2	526 (8%)	189 (8%)	233 (8%)		274 (10%)	145 (9%)	101 (6%)	
	3	39 (1%)	7 (<1%)	18 (1%)		17 (<1%)	10 (<1%)	11 (1%)	
Looking after myself	1	6085 (98%)	2354 (98%)	2768 (99%)	.047	2790 (98%)	1671 (99%)	1535 (98%)	.039
	2	94 (2%)	42 (2%)	27 (1%)		41 (2%)	22 (1%)	30 (2%)	
	3	17 (1%)	3 (<1%)	4 (<1%)		4 (<1%)	3 (<1%)	9 (<1%)	
Doing usual activities	1	5497 (89%)	2192 (92%)	2440 (88%)	<.001	2481 (88%)	1535 (91%)	1393 (89%)	.020
	2	602 (10%)	181 (8%)	311 (11%)		309 (11%)	140 (8%)	150 (10%)	
	3	81 (1%)	19 (<1%)	39 (1%)		40 (1%)	16 (1%)	25 (1%)	
Having pain or discomfort	1	4339 (70%)	1769 (74%)	1902 (68%)	<.001	1942 (69%)	1182 (70%)	1148 (73%)	.044
	2	1671 (27%)	580 (24%)	803 (29%)		805 (28%)	464 (27%)	382 (24%)	
	3	170 (3%)	44 (2%)	89 (3%)		82 (3%)	44 (3%)	41 (3%)	
Feeling worried, sad or unhappy	1	3717 (60%)	1752 (74%)	1338 (48%)	<.001	1810 (64%)	1097 (65%)	750 (48%)	<.001
	2	2027 (33%)	560 (23%)	1169 (42%)		832 (30%)	505 (30%)	666 (42%)	
	3	422 (7%)	76 (3%)	280 (10%)		174 (6%)	89 (5%)	153 (10%)	

In comparison to the first four questions which focus on physical health, the final question around ‘feeling worried, sad or unhappy’ had a higher percentage of pupils responding as having a high level of difficulties (1–3% for the first four questions vs 7% for the emotional question) (Table 7). When results were split by gender and by year group, both male and female pupils across all year groups scored in the higher difficulties range for the emotion related question.

There was a higher percentage of female pupils responding as feeling ‘very worried, sad or unhappy’ compared to male pupils (10% vs 3%). There was a higher percentage of Year 11 pupils responding as feeling ‘very worried, sad or unhappy’ compared to Year 8 and Year 9 pupils (10% vs 6% vs 5%).

### Social media

46% of pupils thought that social media had a positive effect on their overall mood, compared to only 6% who thought social media had a negative effect on their overall mood. 41% responded as social media having neither a positive or negative overall effect, and the remaining 7% did not complete this question.

Of those 46% of pupils who thought that social media had a positive impact on their mood, 5% reported they were feeling ‘very worried, sad or unhappy’ on the final well-being question on the EQ-5D-Y. In comparison, of the 6% of pupils who felt that social media had a negative impact on their mood, 20% reported they were feeling ‘very worried, sad or unhappy’.

The second question asked pupils to rate how much they felt using social media had impacted their mood over the past week. Overall, 44% of pupils responded as ‘not at all’, 38% responded with ‘some of the time’, 11% responded with ‘more than half the time’ and 4% responded with ‘all of the time’. The remaining 4% did not complete this question. When scores were split according to whether the pupils reported social media as having either a positive or negative effect on their mood for the first question, the impact scores remained similar (Table 8).

**Table 8.** Social media impact.

Over the past week, how much has social media affected your mood?	Social media has an overall positive effect on mood (%)	Social media has an overall negative effect on mood (%)
Not at all	36	27
Some of the time	42	47
More than half the time	15	19
All of the time	6	5
Not completed	1	1

### Resource use

A bespoke resource use questionnaire was completed by the pupils. This asked them to record the number and type of services they had accessed in the past 12 months. General health services were the most commonly accessed, with over 57% of pupils having accessed at least one service in the past 12 months. 26% of pupils recorded having accessed a school-based service (e.g. school nurse, school well-being worker or school counsellor), and 6% had accessed specific mental health services.

There were some inconsistencies in the way the forms were completed. For example, although the form asked them to record the number of appointments, approximately 2464 pupils (39%) ticked. Where a pupil had ticked a resource instead of recording a number, this was given a value of 1 appointment. Therefore, although the resource use analysis can be used to give an overview of overall patterns, results should be interpreted with caution.

179 (2.8%) pupils recorded that they used a helpline over the past 12 months. The number of recorded calls for each pupil ranged from 1 to 12, with a total number of approximately 269 calls recorded overall. 230 (3.6%) pupils recorded visiting either a Psychiatrist or a Clinical Psychologist within the past 12 months. There were 415 recorded visits to a Mental Health Nurse, from 197 (3.1%) pupils. 474 (7.5%) pupils reported accessing either a CAMHS therapist, a family therapist or a primary mental health worker (PMHW).

In terms of school-based services, after appointments with a school nurse, the most commonly used services were well-being workers (education-based mental health workers) (6.5% of pupils had accessed) and school counsellors (6.2% of pupils had accessed). Pupils were given space to record any other support they had used through school. These commonly included teachers, teaching assistants and heads of year or heads of house.

In terms of mental health service use in the past year, of those pupils that scored ‘very high’ on the SDQ total difficulties scale, 24% had accessed one of these services at least once. This suggests that almost three quarters of pupils who are identified as in need of extra mental health support have not accessed these services. School based service use was high for this group, with 42% having accessed this.

## Discussion

This study provides an overview of the mental health and well-being scores collected from over 6000 pupils at secondary schools in the North of England. Previous population-based studies exploring mental health in the United Kingdom have had the following approximate numbers in the same age group; Meltzer and colleagues (2000) completed 4525 interviews with 11–15 year olds, McGinnity and colleagues (2005) had 3344 completed interviews with 11–16 year olds and the most recent report had 2609 completed interviews with 11–16 year olds (Sadler et al., 2018). This is therefore one of the largest UK studies of this kind over the last 20 years in terms of young people self-report measures.

The findings show that overall there are large numbers of pupils scoring highly on difficulties measures, particularly for emotional problems. Female pupils scored higher than male pupils on emotional difficulties measures and male pupils scored higher than females on conduct and hyperactivity measures. When split by year group, older pupils in year 11 scored higher than year 8 and 9 pupils for emotional difficulties and prosocial problems.

The recent results for NHS Digital (Sadler et al., 2018) report that one in seven (14.4%) of 11–16 year olds in 2017 had a mental disorder, and that girls and boys were equally likely to experience a disorder. Emotional disorders were the most common, with 9% of 11–16 year olds experiencing this and higher rates for girls than boys. 6.2% of young people had a behavioural disorder, with boys scoring higher than girls. 2.0% of 11–16 year olds experienced a hyperactivity disorder, with boys scoring higher than girls. Similar results were found in our survey, with 15% of pupils scoring within a ‘high’ or ‘very high’ range for total difficulties on the self-report SDQ, and higher scores on emotional subscales for girls and higher rates on behavioural subscales for boys.

Previous to this 2018 report, the most up-to-date official figures were collected in 2004 (McGinnity et al., 2005) comparing figures from 1999 to 2004. In 1999 and in 2004 for the 11- to 15-year-old age range, there was a higher percentage of girls with an emotional disorder compared to boys and a higher percentage of boys with conduct disorders. This shows the gender split in terms of type of disorders and problems faced by young people has remained consistent over time.

Over time, the results from these surveys suggest an increase in mental health problems in this age group, with a prevalence rate of any disorder in 11–15 year olds reported as 11.2% in 1999 (Meltzer et al., 2000), to 11.7% in 2004 (McGinnity et al., 2005) and 14.4% of 11–16 year olds having a mental disorder in 2017 (Sadler et al., 2018). Our study, conducted in 2018, although it does not present numbers of children with diagnosed disorders, shows a similar trend with approximately 15% of pupils aged 12–16 years old scoring ‘high’ or ‘very high’ on measures of overall difficulties. Our study does not have the information to understand the specific reasons for this increase. Deighton and colleagues (2019) highlight factors that are associated with differences in young people’s mental health such as sex, age, ethnicity, deprivation and children in need status. Research has shown that a range of other factors should be considered including academic pressures, family relationships, environment and lifestyle (Evans & Hurrell, 2016; Sweeting et al., 2010). Our study shows 76% of young people with apparent significant mental health problems appear to receive no external mental health service support at all. This needs to be treated with caution given that this figure does not include school-based services and general health services which may have been accessed for mental health support in this group.

The results from the current survey suggest that more targeted work is necessary. For example, EQ-5D-Y results highlight the gender difference between pupils, with 10% of girls reporting as feeling ‘worried, sad or unhappy’, compared to 3% of boys. This is at odds with the Sadler and colleagues (2018) study which suggests that male and female pupils were equally likely to have a disorder (14.3% for males and 14.4% for females). However, Sadler suggests that there is a gender difference in terms of type of disorder, with girls more likely to experience emotional disorders and boys more likely to experience behavioural disorders. The EQ-5D-Y results reflect emotional disorders more than conduct disorders. The EQ-5D-Y also shows a much higher percentage of year 11 pupils reporting feeling ‘worried, sad or unhappy’ compared to year 8 and year 9 pupils. There were similar findings for the SDQ scales, with results suggesting that older children (specifically year 11 pupils) were more likely to score highly on difficulty measures compared to younger children. Pupils face a range of challenges throughout year 11 including examinations, school pressures and making decisions around their future after leaving school. We did not directly test these hypotheses through our survey and so future work should consider investigating the factors which contribute to the higher levels of difficulties for these pupils allowing more targeted support to be offered.

This survey provides initial findings showing how young people mainly view the use of social media as having a positive impact on their mood. Recent studies have linked high social media use to online harassment, poor sleep and self-esteem problems (Booker et al., 2018). It is important to recognise and explore how social media can be used in both positive and negative ways for this age group, as well as identifying the role of schools in helping young people use social media safely (Daine et al., 2013; Marchant et al., 2017).

The resource use data collected in this survey between the academic year 2017 and 2018 shows that a high proportion of pupils are accessing mental health support through schools, with one-quarter of pupils who completed questionnaires reporting having used at least one school-based service. Increasingly, schools are becoming a place where children and young people feel they are able to access mental health support, highlighting the importance of the Government’s Mental Health green paper (Department of Health and Social Care and Department for Education, 2017) plans to focus on and improve service provision in school settings. Further research in this area evaluating the roll out of new school-based services will be key to test effectiveness.

As this study was conducted with limited resources, there were some limitations faced. Only pupil self-report SDQs were completed. Multi-informant SDQs allow more comprehensive results to be collected. Another potential limitation is that it is possible that as schools chose whether to participate, the final sample may not be fully representative. Despite this, our sample of participating schools was diverse in terms of socioeconomic and geographical factors. Further research could address these limitations; by collecting multi-informant SDQs and details around why some schools choose not to participate.

Results from our survey show that relatively high numbers of young people are facing mental health problems, and these have possibly been increasing over time. In the North of England, it is a current issue, faced particularly by female pupils and older pupils in year 11. There is a need for early, targeted interventions, which can be delivered and accessed by pupils in schools. It is important that up-to-date measures are collected around the mental health and well-being of this group, to allow for planning of services and earlier intervention to support those facing difficulties.
